# CMV-IgG pre-allogeneic hematopoietic stem cell transplantation and the risk for CMV reactivation and mortality

**DOI:** 10.1038/s41409-023-01944-2

**Published:** 2023-03-03

**Authors:** Kirsten Alexandra Eberhardt, Verena Jung, Elena Knops, Eva Heger, Maike Wirtz, Gertrud Steger, Rolf Kaiser, Patrick Affeldt, Udo Holtick, Florian Klein, Christof Scheid, Veronica Di Cristanziano

**Affiliations:** 1 Division of Hygiene and Infectious Diseases, Institute of Hygiene and Environment, Hamburg, Germany; 2 Department of Tropical Medicine, Bernhard Nocht Institute for Tropical Medicine & I. Department of Medicine, University Medical Center Hamburg-Eppendorf, Hamburg, Germany; 3 Department of Hematology and Stem-Cell Transplantation, University Hospital Essen, Essen, Germany; 4 Department I of Internal Medicine, Faculty of Medicine and University Hospital Cologne, University of Cologne, Cologne, Germany; 5 Institute of Virology, Faculty of Medicine and University Hospital Cologne, University of Cologne, Cologne, Germany; 6 Department II of Internal Medicine and Center for Molecular Medicine Cologne, Faculty of Medicine and University Hospital Cologne, University of Cologne, Cologne, Germany

**Keywords:** Risk factors, Infectious diseases

## Abstract

Cytomegalovirus (CMV) represents one of the most common infectious complications after allogeneic hematopoietic stem cell transplantation (allo-HSCT). Currently, a common diagnostic test used to stratify the risk for CMV infection in allo-HSCT recipients is the qualitative CMV serology of donor and recipient. A positive serostatus of the recipient is the most important risk factor for CMV reactivation and associated with reduced overall survival post-transplantation (TX). Direct and indirect effects of CMV are involved in the poorer survival outcome. The present study investigated if the quantitative interpretation of anti-CMV IgG before allo-HSCT might serve as a novel parameter for the identification of patients at risk for CMV reactivation and worse outcome post-TX. For this purpose, a cohort of 440 allo-HSCT recipients over a period of 10 years was retrospectively analyzed. Our findings indicated that patients with high CMV IgG pre-allo-HSCT had a higher risk to develop CMV reactivation, including clinically relevant infections, and a worse prognosis 36 months post-allo-HSCT as compared to recipients with low CMV IgG values. In the letermovir (LMV) era, this group of patients might benefit from a closer CMV monitoring, and hence, earlier intervention if needed, especially after discontinuation of prophylaxis.

## Introduction

Cytomegalovirus (CMV) represents one of the most important opportunistic infections after allogeneic hematopoietic stem cell transplantation (allo-HSCT) [1, 2]. Despite relevant advances in diagnostics and therapy, CMV continues to adversely affect the clinical outcome in this group of vulnerable patients. The risk of CMV infection after allo-HSCT varies according to the serostatus of donor (D) and recipient (R). CMV infections following allo-HSCT are mostly the consequence of viral reactivation in seropositive recipients (R + ) [3]. Up to 80% of CMV seropositive recipients can experience viral reactivation after allo-HSCT, with the CMV discordant serostatus (D-/R + ) associated with the highest risk for severe CMV disease [4, 5].

Besides being one of the most important risk factors for CMV reactivation and disease, a CMV positive serostatus of the recipient pre-transplantation (TX) is known to be an independent risk factor for poor outcome after allo-HSCT [6, 7]. The negative impact of CMV includes direct effects mediated by viral replication and indirect biological effects induced by viral immunomodulatory properties [8]. CMV reactivation is reported to be associated with an increased risk for secondary bacterial and fungal infections, as well as graft-versus-host-disease (GvHD) [1, 6, 9, 10]. Furthermore, most of available anti-CMV agents are characterized by relevant drug toxicity, which also impacts negatively on the recovery post-TX [11].

Two main strategies are available for CMV prevention in allo-HSCT recipients. In case of preemptive therapy, patients are closely monitored after TX by real-time PCR to detect early CMV replication with the initiation of antiviral therapy upon detection of CMV-DNA in blood at a predetermined threshold. Before availability of letermovir (LMV), this approach was preferred in allo-HSCT recipients to avoid myelotoxicity of conventional antiviral agents. Since LMV was approved in 2017, antiviral prophylaxis has become an option also in the hematological setting [12]. The advent of preventive strategies has drastically reduced the occurrence of CMV disease in allo-HSCT recipients from 30% to less than 5% [11]. Antiviral prophylaxis can be associated to late-onset CMV infection following LMV discontinuation, whereas the preemptive approach is still missing a universal threshold of viral load for guiding antiviral therapy [8, 13].

CMV has a negative impact on patient outcome and a CMV positive serostatus is an independent risk factor for non-relapse mortality as shown by consistent data [7, 14]. In line with these observations, prevention remains crucial to protect transplant recipients by the adverse effects of CMV. Thus, the identification of new diagnostic tools able to individualize risk stratification are urgently needed to optimize antiviral drug exposure and intensity of immunosuppressive regimens [15].

Currently, the pre-TX assessment of D and R serostatus is the most common laboratory assay to stratify the risk for CMV after allo-HSCT. In the post-transplant period, the monitoring of CMV cell-mediated immunity by interferon-γ release assay (IGRA) is increasingly recognized as a valid tool to assess the ability of patient immune response of controlling viral replication [16, 17].

The detection of anti-CMV IgG is an important qualitative biomarker for the definition of latent CMV infection in recipients and donors, whereas the relevance of anti-CMV IgG as quantitative value has been poorly considered. The few available data suggest that the quantitative determination of specific anti-CMV IgG could represent an early parameter of CMV risk assessment in candidates for allo-HSCT [17, 18].

In the present retrospective analysis, we investigated if the pre-allo-HSCT anti-CMV IgG value, measured by a worldwide used commercially automated assay, might serve as a novel parameter to identify patients at higher risk for CMV reactivation and, eventually, poorer outcome post-TX.

## Material and methods

### Patients

For this analysis, we included retrospective data of adult patients undergoing allo-HSCT at the University Hospital of Cologne between February 2008 and April 2019 with available pre-TX CMV serology performed at our laboratory. Patients were actively followed up until June 2020. Exclusion criteria were CMV viremia before HSCT, a previous HSCT, missing data on CMV-DNA monitoring post-allo-HSCT, or lost to follow-up. 440 patients (185 females and 255 males) with a median age of 49.5 years, were included into this analysis. The conditioning regimen used prior to the allo-HSCT consisted of 335 (76.14%) reduced intensity conditioning (RIC), 86 (19.55%) myeloablative conditioning (MAC) and 19 (4.32%) non-myeloablative (NMA). Allo-HSCT was performed due to mostly malignant diseases (99.09%), of which 211 (48.39%) were classified with intermediate risk, 126 (28.9%) with high, 62 (14.22%) with low and 37 (8.49%) with very high risk, according to the disease risk index (DRI) [19]. Of the stem cell transplants that were used, 332 (75.45%) were of matched related or unrelated donors, while 108 (24.55%) were mismatched.

CMV serology was timely performed in all donors and recipients before allo-HSCT. The CMV serostatus before TX was defined as seronegative when donor or recipient sample was not reactive for anti-CMV IgG and seropositive in case of reactivity for anti-CMV IgG, as described below.

According to the preemptive approach used in our center, allo-HSCT recipients (R + and R-) were screened for CMV-DNA load in whole blood samples by real-time PCR twice a week for the first 100 days, and once weekly for the following twelve months after allo-HSCT. Based on our internal standard, preemptive antiviral therapy (mainly valganciclovir or i.v. ganciclovir) was initiated upon detection of a CMV-DNA load once > 2000 IU/ml or twice > 1000 IU/ml. Clinically significant CMV infection (csCMVi) was defined as detection of CMV DNAemia requiring the start of preemptive medication, according to our center treatment protocol [20]. CMV DNAemia not necessitating antiviral treatment was defined as subclinical CMV infection (subCMVi).

Patients were categorized by means of their CMV-serological status into four possible groups. A combination of the CMV serostatus of donor and recipient revealed the following groups: D + /R + , D + /R-, D-/R + and D-/R-. The study was conducted in compliance with the Declaration of Helsinki and according to the protocol approved by the Ethics Committee of the Medical Faculty of the University of Cologne, Germany (08-160). All patients provided informed consent.

### CMV serology for the measurement of anti-CMV IgG

Anti-CMV IgG were detected using the Abbott Architect CMV IgG chemiluminescent microparticle immunoassay (CMIA) [21, 22]. The assay uses a human CMV lysate (strain AD 169) and is performed on the automated platform Abbott Architect i2000SR (Abbott, Abbott Park, IL, United States). The CMV IG assay provided by Abbott is a two-step immunoassay for qualitative and semiquantitative measurement of anti-CMV IgG. Sample reactivity is determined by comparing the chemiluminescent signal of samples to the cut-off signal of calibration and results are reported in AU/ml (arbitrary unit per milliliter). The calibration range is between 0 and 250 AU/ml. According to manufacturer instructions, sample values ≥ 6 AU/mL were interpreted as reactive for anti-CMV IgG. Reactive samples with IgG values ≥250 AU/ml were not further diluted. In our routine diagnostics, all patients requiring CMV serology pre-TX are tested for both, anti-CMV IgM and IgG. In case of simultaneously positive detection of anti-CMV IgM and IgG, samples are retested for determination of CMV IgG avidity. Only in this case, samples with IgG values ≥250 AU/ml are automatically retested in 1:10 dilution, according to manufacturer’s instructions.

For the present analysis, recipients were stratified into three groups (A-C), according to the detected pre-TX anti-CMV IgG value. More in detail, CMV seropositive patients with anti-CMV IgG values ≥250 AU/ml were defined as group A, whereas patients with anti-CMV IgG values between 6 and 249 AU/ml were assigned to group B. Seronegative recipients (anti-CMV IgG value 0–5 AU/ml) were defined as group C.

### Statistical analyses

Continuous variables were expressed as mean ± standard deviation (SD) or median (interquartile range, IQR) and compared using ANOVA or the Kruskal-Wallis test. Categorical variables were compared using either the χ 2 test or the Fisher exact test, as appropriate. In case of post-hoc tests, FDR correction for multiple testing was applied. Associations of independent parameters were assessed by using multiple ordinal and linear regression models adjusting for covariates. Cox proportional-hazard models were applied using the “survminer” package in R (version 4.0.5, R Foundation for Statistical Computing, Vienna, Austria, URL https://www.Rproject.org/, the code supporting the conclusions of this article can be made available upon request to the corresponding author) and the “cutpointr” package was employed for finding the appropriate anti-CMV IgG value as cut-off for the definition of patient groups. Two-sided *p* values were presented, and an α of 0.05 was determined as significant.

## Results

### Characteristics and outcomes according to donor and recipient qualitative CMV IgG serostatus

A total of 440 allo-HSCT patients were retrospectively analysed for this study and grouped according to the pre-TX CMV serostatus of donor and recipient (Table 1). The largest group (*n* = 175, 39.8%) included patients with a seropositive donor and recipient combination (D + /R + ). One third (30.7%) of patients were seronegative and received hematopoietic stem cells of a seronegative donor. 20.9% of the cohort consisted of seropositive recipients with a seronegative donor. The smallest group were seronegative patients who received hematopoietic stem cells from a CMV-seropositive donor (8.7%). The mean age of participants was 49.5 ± 14.0 years and differed slightly between the groups. Besides that, there were no differences in sex, conditioning, DRI or matching quality. Whereas the proportion of mortality was equally high in all groups, the rate of CMV infections was highest in seropositive recipients (Table 1).

**Table 1:** Characteristics and outcomes according to donor and recipient qualitative CMV IgG serostatus.

Cohort	All patients	D-/R +	D + /R +	D + /R-	D-/R-	*p* value
n (%)	440	92 (20.91)	175 (39.77)	38 (8.64)	135 (30.68)	
Age in years ± SD	49.5 ± 14.0	49.9 ± 12.7	51.9 ± 14.6	43.0 ± 13.8	47.8 ± 13.3	0.001
Female, n (%)	185 (42.05)	45 (48.91)	75 (42.86)	17 (44.74)	48 (35.56)	0.233
Conditioning, n (%)						0.795
MAC	86 (19.55)	17 (18.48)	35 (20.00)	11 (28.95)	23 (17.04)	
NMA	19 (4.32)	4 (4.35)	7 (4.00)	1 (2.63)	7 (5.19)	
RIC	335 (76.14)	71 (77.17)	133 (76.00)	26 (68.42)	105 (77.78)	
Disease risk, n (%)						0.227
Low	62 (14.22)	17 (18.68)	25 (14.53)	2 (5.26)	18 (13.33)	
Intermediate	211 (48.39)	39 (42.86)	88 (51.16)	15 (39.47)	69 (51.11)	
High	126 (28.90)	26 (28.57)	44 (25.58)	15 (39.47)	41 (30.37)	
Very high	37 (8.49)	9 (9.89)	15 (8.72)	6 (15.79)	7 (5.19)	
Optimal match vs. mismatch, n (%)	332 (75.45)	67 (72.83)	131 (74.86)	28 (73.68)	106 (78.52)	0.771
CMV infection event, n (%)						<0.001
No infection	212 (48.18)	23 (25.00)	28 (16.00)	32 (84.21)	129 (95.56)	
subCMVi	74 (16.82)	14 (15.22)	55 (31.43)	2 (5.26)	3 (2.22)	
csCMVi	154 (35.00)	55 (59.78)	92 (52.57)	4 (10.53)	3 (2.22)	
Death, n (%)	234 (53.55)	58 (63.04)	84 (48.28)	21 (55.26)	71 (52.99)	0.149

D – donor.

R – recipient.

+ – CMV seropositive.

- – CMV seronegative.

*MAC* myeloablative conditioning, *NMA* non-myeloablative, *RIC* reduced intensityconditioning, *subCMVi* subclinical infection, *csCMVi* clinically significantinfection.

### Characteristics and outcomes according to recipient quantitative anti-CMV IgG value

**Fig. 1:**
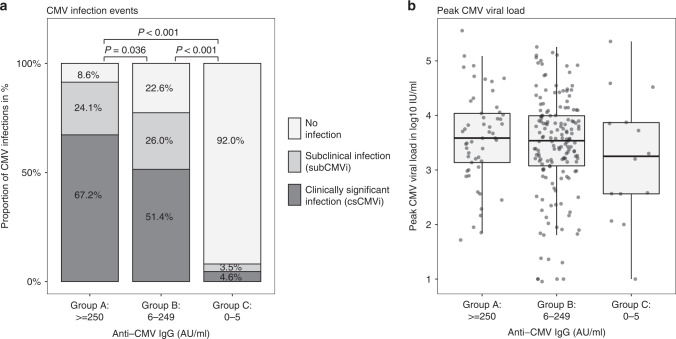
Occurrence of CMV infections after allogeneic hematopoietic stem cell transplantation (allo-HSCT) according to pre-transplantation (pre-TX) anti-CMV IgG values. **a** The proportion of CMV infections, including no infection, subclinical (subCMVi), and clinically significant CMV infections (csCMVi), were compared between three different patient groups (A, B, C), according to pre-TX anti-CMV IgG values. P values are presented for pairwise post hoc comparisons. **b** CMV DNA peak viral loads (subCMVi and csCMVi) were compared between three different patient groups (A, B, C), according to pre-TX anti-CMV IgG values. *P* values are presented for pairwise post hoc comparisons. AU/ml arbitrary units per milliliter.

CMV seropositive recipients with a pre-TX anti-CMV IgG of 250 AU/ml or higher (group A) did not differ with regards to demographic parameters, the proportion of seropositive donors, medical treatment, disease risk, or matching quality from group B (anti-CMV IgG 6-249 AU/ml, Table 2). CMV infections after allo-HSCT occurred in a larger proportion of patients within group A compared to group B and C. In detail, 24.14% of group A patients developed a subCMVi and 67.24% a csCMVi, whereas 26% of group B individuals were diagnosed with a subCMVi and 51.44% with a csCMVi (*p* = 0.036, Fig. 1a). However, peak CMV viral loads post-TX, as well as the median duration of CMV reactivation was not statistically different between group A and B (Fig. 1b). The proportion of deaths over the entire observation period was significantly higher in patients with high pre-TX anti-CMV IgG values (group A) compared to patients from group B with IgG values between 6 and 249 AU/ml or group C with values between 0 and 5 AU/ml (74.14% vs. 47.34%, *p* = 0.002 and vs. 53.76%, *p* = 0.015, Table 2).

**Table 2:** Characteristics and outcomes according to recipient quantitative anti-CMV IgG value.

CMV seropositive recipients	Group A: anti-CMV IgG ≥250 AU/ml	Group B: anti-CMV IgG 6–249 AU/ml	Group C: anti-CMV IgG 0–5 AU/ml	*p* value	Post-hoc *p* value A/B	Post-hoc *p* value A/C	Post-hoc *p* value B/C
n (%)	58 (13.18)	208 (47.27)	174 (39.55)	–	–	–	–
Age in years ± SD	53.8 ± 12.8	50.5 ± 14.2	46.7 ± 13.5	0.001	0.122	0.001	0.007
Female, n (%)	27 (46.55)	93 (44.71)	65 (37.36)	0.264			
Donor CMV seropositive, n (%)	36 (62.07)	136 (65.38)	41 (23.56)	<0.001	0.755	<0.001	<0.001
Conditioning, n (%)				0.993	–	–	–
MAC	11 (18.97)	41 (19.71)	34 (19.54)				
NMA	3 (5.17)	8 (3.85)	8 (4.60)				
RIC	44 (75.86)	159 (76.44)	132 (75.86)				
Disease risk, n (%)					–	–	–
Low	11 (19.30)	31 (15.12)	20 (11.49)				
Intermediate	22 (38.60)	105 (51.22)	84 (48.28)	0.149			
High	21 (36.84)	48 (23.41)	57 (32.76)				
Very high	3 (5.26)	21 (10.24)	13 (7.47)				
Optimal match vs. mismatch, n (%)	41 (70.69)	156 (75.00)	135 (77.59)	0.560	–	–	–
CMV infection event, n (%)				<0.001	0.036	<0.001	<0.001
No infection	5 (8.62)	47 (22.60)	160 (91.95)				
subCMVi	14 (24.14)	54 (25.96)	6 (3.45)				
csCMVi	39 (67.24)	107 (51.44)	8 (4.60)				
CMV peak viral load in IU/ml (IQR)	3840.0 (1370.0–10900.0)	3440.0 (1190.0–9870.0)	1795.0 (364.8–7390.0)	0.551	–	–	–
Days of CMV reactivation (IQR)	78.5 (33.2–147.0)	88.0 (38.0–161.0)	12.5 (7.0–119.8)	0.018	0.400	0.042	0.017
Days between TX and CMV reactivation (IQR)	39.0 (25.0–52.5)	30.5 (24.0–42.0)	47.0 (37.0–116.0)	0.031	0.150	0.195	0.072
Death, n (%)	43 (74.14)	98 (47.34)	93 (53.76)	0.001	0.002	0.015	0.253

*MAC* myeloablative conditioning, *NMA* non-myeloablative, *RIC* reduced intensityconditioning, *subCMVi* subclinical infection, *csCMVi* clinically significantinfection, *IQR* interquartile range.

**Fig. 2:**
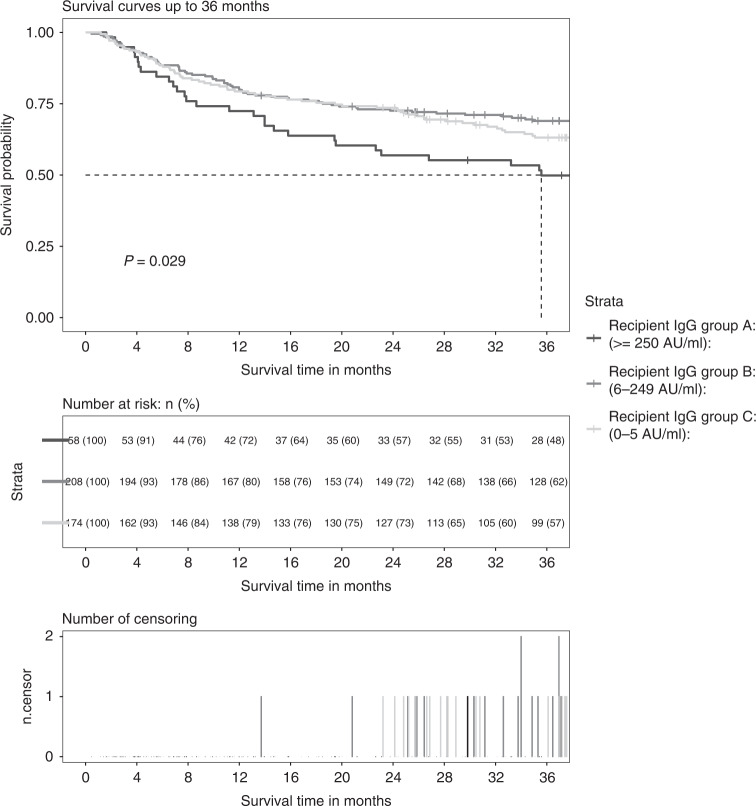
Kaplan–Meyer survival curves, number at risk and number of censoring after allogeneic hematopoietic stem cell transplantation (allo-HSCT) according to pre-transplantation (pre-TX) anti-CMV IgG values. Displayed are survival curves (top) up to 36 months post-allo-HSCT according to pre-TX anti-CMV IgG values in allo-HSCT recipients (group A, B and C) with numbers at risk (middle) and number of censoring (bottom). The *P* value represents the result of the logrank test between the survival curves of the three groups. AU/ml arbitrary units per milliliter.

Overall survival of the present cohort was 79% at 12 months and 64% at 36 months post-allo-HSCT. The Kaplan Meyer survival curves demonstrated significant differences in survival time between the groups for the first 36 months (*p* = 0.029, logrank test, Fig. 2) but not for the first 12 months after allo-HSCT. It was also noted that the number of early deaths within the first months was low in all groups (Supplementary Figure). In the multiple cox proportional-hazards model adjusting for covariates, a pre-TX anti-CMV IgG below 250 AU/ml (group B and C) was associated with prolonged time to death compared to patients with higher pre-TX anti-CMV IgG values in the first 36 months (aHR 0.58 [0.37, 0.91 95%CI] and aHR 0.62 [0.39, 1.01] respectively, Table 3) but not in the first 12 months after allo-HSCT. Survival times of group B and C (pre-TX IgG 6-249 and 0-5 AU/ml) were not different 12 and 36 months after allo-HSCT (Supplementary Table). Only few early deaths within the first months post-TX occurred in the entire cohort. In addition, a lower pre-TX anti-CMV IgG value was significantly associated with less CMV infection events, as shown by the multiple ordinal regression model in Table 4 (aOR 0.51 [0.27, 0.92] and 0.02 [0.01, 0.03]).

**Table 3:** Factors associated with time to death.

Dependent variable	a. Cox proportional−hazards model up to 12 months		b. Cox proportional−hazards model up to 36 months	
	Adjusted hazard ratio	*p* value	Adjusted hazard ratio	*p* value
Age in years	1.01 (1.00, 1.03)	0.099	1.01 (0.99, 1.02)	0.371
Recipient anti−CMV IgG group				
A ( = 250 AU/ml)	1		1	
B (6 − 249 AU/ml)	0.77 (0.42, 1.41)	0.397	0.58 (0.37, 0.91)	0.017
C (0 − 5 AU/ml)	0.78 (0.41, 1.47)	0.434	0.62 (0.39, 1.01)	0.053
Donor CMV serostatus				
Positive	1		1	
Negative	1.22 (0.77, 1.92)	0.396	1.30 (0.91, 1.85)	0.144
Disease risk				
Low	1		1	
Intermediate	1.18 (0.56, 2.49)	0.658	1.58 (0.88, 2.84)	0.122
High	2.29 (1.09, 4.80)	0.028	2.54 (1.41, 4.58)	0.002
very high	2.85 (1.19, 6.85)	0.019	2.78 (1.35, 5.76)	0.006
Match				
Match	1		1	
Mismatch	1.33 (0.83, 2.13)	0.233	1.16 (0.80, 1.68)	0.436
Conditioning				
MAC	1		1	
NMA	1.63 (0.53, 5.03)	0.3990	2.01 (0.85, 4.77)	0.113
RIC	1.19 (0.68, 2.09)	0.541	1.43 (0.91, 2.24)	0.123

**Table 4:** Factors associated with CMV infection.

Dependent variable	a. CMV infection events		b. Peak CMV viral load	
	Multiple ordinal regression model		Multiple linear regression model	
	Adjusted odds ratio	*p* value	Adjusted ß-estimates	*p* value
Age in years	1.02 (1.01, 1.04)	0.001	0.00 (−0.01, 0.01)	0.873
Recipient anti−CMV IgG group				
A ( = 250 AU/ml)	1		1	
B (6 − 249 AU/ml)	0.51 (0.27, 0.92)	0.030	0.02 (−0.26, 0.30)	0.891
C (0 − 5 AU/ml)	0.02 (0.01, 0.03)	<0.001	−0.33 (−0.85, 0.19)	0.216
Donor CMV serostatus				
Positive	1		1	
negative	0.75 (0.47, 1.20)	0.223	0.48 (0.23, 0.72)	<0.001
Disease risk				
Low	1		1	
Intermediate	0.71 (0.37, 1.35)	0.294	0.37 (−0.71, −0.04)	0.030
High	0.80 (0.39, 1.64)	0.548	−0.08 (−0.45, 0.30)	0.685
Very high	0.93 (0.37, 2.32)	0.875	−0.44 (−0.92, 0.04)	0.073
Match				
Match	1		1	
Mismatch	1.08 (0.64, 1.80)	0.782	0.24 (−0.03, 0.51)	0.083
Conditioning				
MAC	1		1	
NMA	0.38 (0.11, 1.30)	0.123	−0.76 (−1.43, −0.08)	0.028
RIC	0.53 (0.29, 0.93)	0.298	−0.21 (−0.50, 0.09)	0.165

In contrast to this, a negative donor CMV serostatus but not the recipient pre-TX anti-CMV IgG value was associated with the extent of post-TX viral load, as demonstrated by a multiple linear regression model (Table 4).

## Discussion

After primary infection, CMV establishes a lifelong latent infection in the host under control of immune response. CMV reactivation is a common event in allo-HSCT recipients. In the present study, CMV DNAemia was detected in 52% (228/440) of recipients during the follow-up post-TX. In 95% of cases, CMV reactivated in recipients with a positive CMV serostatus before allo-HSCT, confirming the positive serostatus of the recipient to be the most important risk factor for viral reactivation [23].

Over the last years, the monitoring of CMV T cell reactivity was proposed as a valid tool to guide preemptive treatment in the follow-up post-allo-HSCT, avoiding unnecessary use of antiviral drugs for patients with a strong cellular immune response to CMV, or to optimize the duration of LMV prophylaxis [24]. In turn, CMV serology remains the only test available in the pre-TX period to stratify the risk of CMV occurrence post-TX.

Recent investigations by Arcuri et al. (2020) and Kawamura et al. (2021) evidenced that the quantitative anti-CMV IgG value pre-allo-HSCT is associated with the risk for CMV reactivation after TX. In both studies, a higher anti-CMV IgG titer correlated with a higher risk of CMV reactivation [18, 25]. In line with these previous findings, we observed that patients with anti-CMV IgG values pre-TX ≥ 250 AU/ml (group A) were at significant higher risk of CMV reactivation, including clinically relevant infections, compared to patients with lower IgG values (IgG 6-249 AU/ml, group B). Notable, the quantitative IgG pre-TX value did not correlate with the extent of viral load but with the outcome post-TX. In particular, our data showed that an anti-CMV IgG value pre-TX ≥ 250 AU/ml was associated with a poorer survival 36 months post-allo-HSCT in comparison to lower anti-CMV IgG values, whereas no significant differences in survival were observed in the first 12 months post-allo-HSCT. Previous data demonstrated that CMV infection events are associated to a higher mortality in the first year post-allo-HSCT [14, 26]. In the present cohort, the CMV serostatus was not associated with significant differences in mortality in the early phase post-TX, although an early declining in survival in patients with higher IgG levels was observed. The low number of fatal outcomes in the first months post-TX and the sample size of our cohort might partially explain this finding. Interestingly, no difference in patients with low anti-CMV IgG 6-249 AU/ml (group B) and CMV seronegative (group C) was found in predicting mortality in our study. Therefore, beside the level of CMV replication, we can presume that other viral properties are likely involved in adversely affecting the prognosis of patients with higher antibody values (group A) [27]. Further studies are needed to understand the association between anti-CMV IgG levels pre-TX and late mortality. Furthermore, although the survival of patients with higher IgG and seronegative recipients did not clearly differ at 36 months post-allo-HSCT [23], an anti-CMV IgG value pre-TX of ≥ 250 AU/ml was identified as a significant risk factor for CMV reactivation and worse outcome within the seropositive group.

As known, the T cell response has a crucial role in maintaining CMV in a lifelong state of latency after primary infection [28]. Humoral immunity to CMV is reported to protect against viral dissemination and CMV disease and CMV immunoglobulins can be used in combination to other antiviral agents to treat severe CMV manifestations [4, 28, 29]. Allo-HSCT procedure is followed by a complex process of immune reconstitution and CMV reactivation in the early phase of immune recovery is frequently observed in seropositive recipients [30]. Based on the present results, we hypothesized that the anti-CMV IgG value of the recipient before allo-HSCT could reflect the subclinical activity of viral latent reservoir. It is supposed that CMV can reactivate from latency without being able to be detected in blood. These attempts are normally repressed by the immune response in the immunocompetent host and the constant exposure to viral antigens guarantees the control of the virus. However, the process of immune recovery following allo-HSCT compromises the capacity to contain CMV to resurge from latency and viral replication becomes detectable [31]. This means that higher antibody values may correspond to a more intense interplay between latent CMV and immune adaptive responses, as suggested by the higher risk for reactivation observed in patients with higher pre-TX anti-CMV IgG.

Besides being the most important risk factor for reactivation, a CMV positive serostatus of the recipient is associated with a reduced survival rate [32]. Direct and indirect effects of CMV are considered responsible for the poorer outcome in this group of patients [32, 33]. Also in the present study, direct and indirect effects of CMV are likely to negatively affect patient prognosis in different ways. On the one hand, we found that the quantitative and not the qualitative definition of CMV serostatus pre-TX affected the survival of allo-HSCT recipients at three years post-allo-HSCT, providing new insights in understanding the negative prognostic influence of CMV on transplant recipients [34]. On the other hand, in line with Green et al., a higher CMV viral load post-TX was associated with an increased mortality (p = 0.002, data not shown) [26]. However, the peak viral load in transplant recipients with CMV reactivation in our cohort was not associated with the pre-allo-HSCT anti-CMV IgG value, but with the serostatus of the donor. This finding confirms that a pre-existing anti-viral immunity of the donor is required to control CMV reactivation in seropositive recipients [35–37].

## Conclusions

Early parameters enabling to identify immunocompromised patients at risk of opportunistic infections are highly required. So far, the determination of CMV serostatus in transplant recipients has been used as qualitative parameter. The present study demonstrates a significant association of anti-CMV IgG values pre-allo-HSCT and the risk for CMV reactivation and late mortality post-TX. In the LMV era, this group of patients might benefit from a closer CMV monitoring, and hence, earlier intervention if needed, especially after discontinuation of prophylaxis.

### Limitations

This study has some limitations to mention. Specimens with anti-CMV IgG values of more than 250 AU/ml were flagged as “≥ 250 AU/ml” and were not further diluted to obtain more precise results above 250 AU/ml, considering that so far there was no evidence to perform this additional step routinely. Another potential limitation is that we were reporting serology results in AU/ml. Yet, some assays from different manufacturers are using different units of measurement and a common comparable standard is lacking so far. An additional important limitation to mention is that this study has an explorative, hypothesis forming character and that larger group sizes would have been necessary to detect small effect sizes as defined by Cohen [38]. Therefore, larger prospective multicenter studies are necessary to validate our findings before quantitative pre-TX IgG values can be used as an established predictor in the clinical setting. Furthermore, anti-CMV IgG values of more than 250 AU/ml should be diluted in future studies in order to explore the performance of higher cut-offs and a potential relationship of higher IgG concentrations with peak viral loads.

## Supplementary information

Survival curves up to 12 months

Factors associated with time to death with recipient anti-CMV IgG group C (0-5 AU/ml) as reference group

## Data Availability

The data underlying this article are available in the figshare repository (10.6084/m9.figshare.20343765).
