# Supplementary figures and images for: CMV-IgG pre-allogeneic hematopoietic stem cell transplantation and the risk for CMV reactivation and mortality
Source: Bone Marrow Transplant. 2023 Mar 3;58(6):639–46. doi: 10.1038/s41409-023-01944-2 (PMC10247370; doi:10.1038/s41409-023-01944-2)

Survival curves up to 12 months

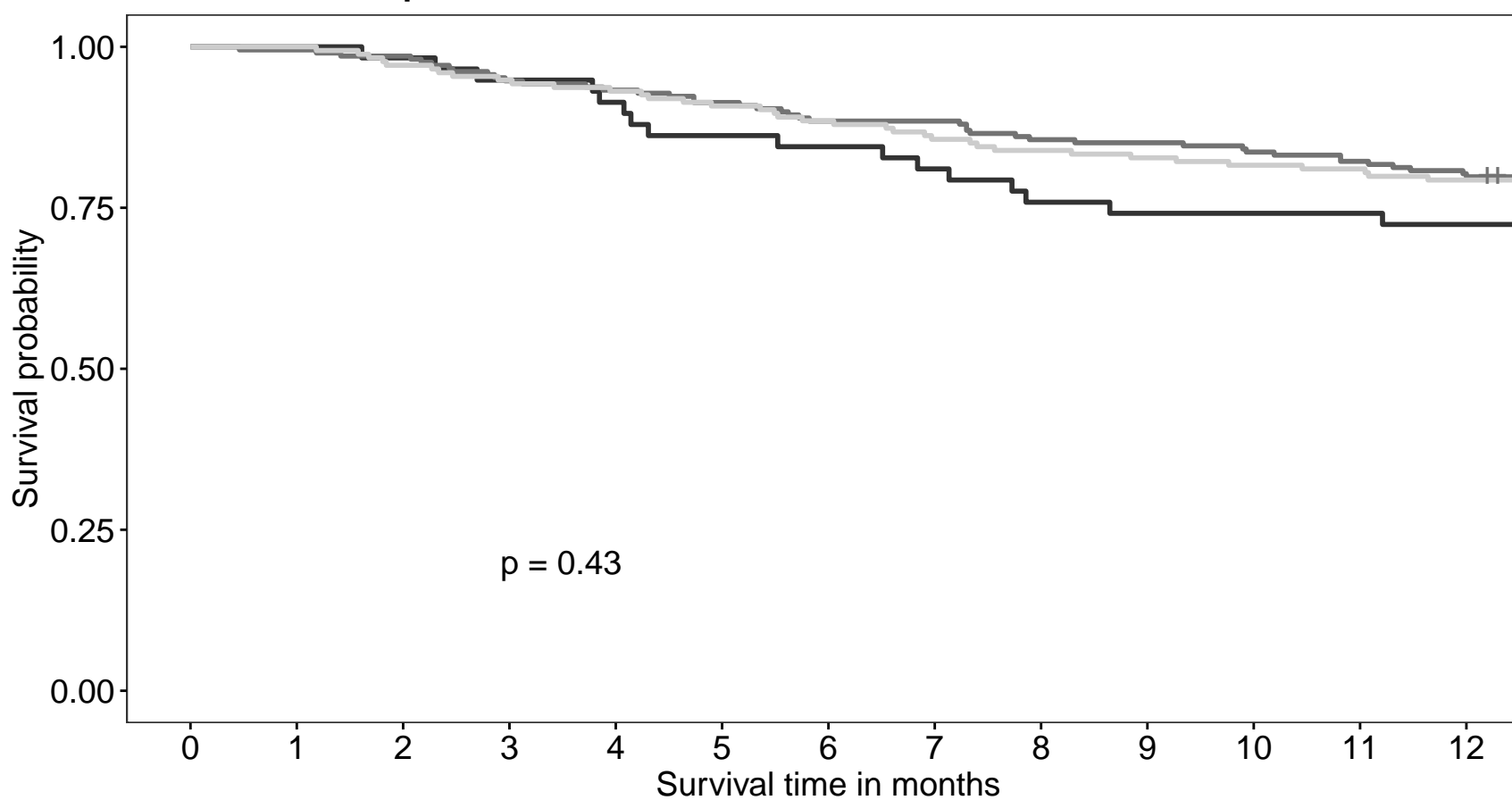

Number at risk: n (%)

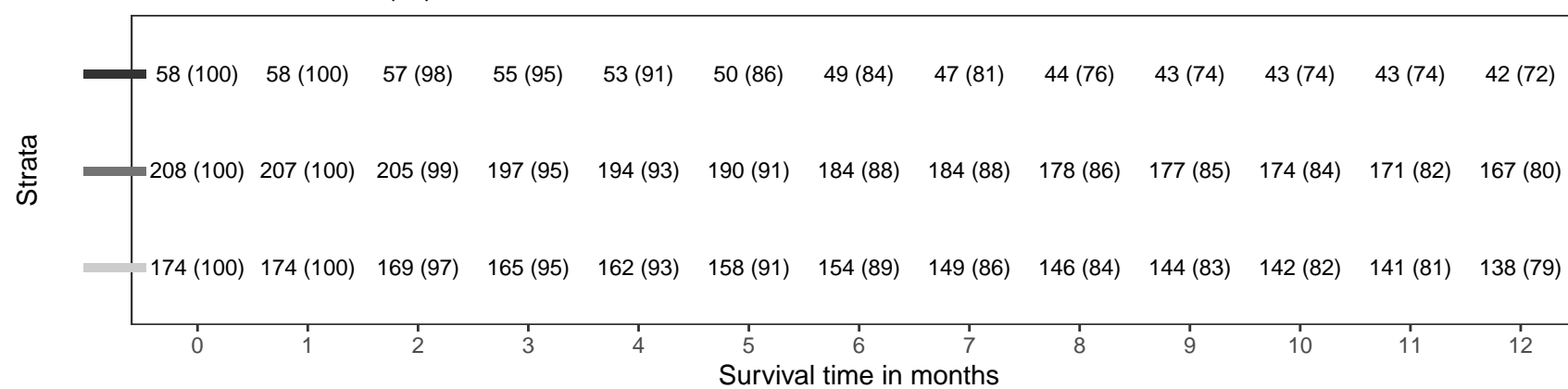

Number of censoring

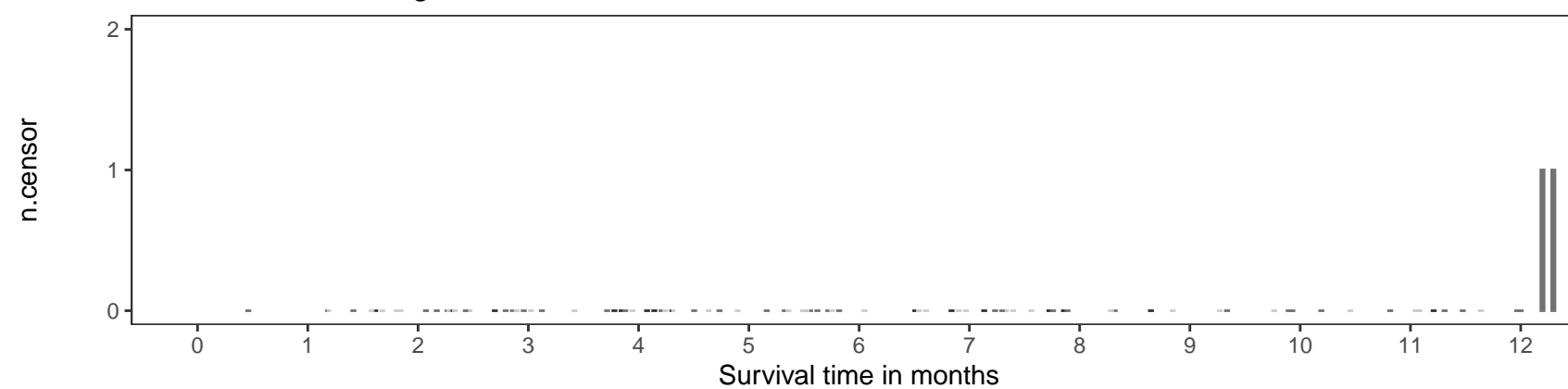

Supplement: Supplementary file 1 — Survival curves up to 12 months [file 41409_2023_1944_MOESM1_ESM.pdf]
