# Supplementary material for: CMV-IgG pre-allogeneic hematopoietic stem cell transplantation and the risk for CMV reactivation and mortality
Source: Bone Marrow Transplant. 2023 Mar 3;58(6):639–46. doi: 10.1038/s41409-023-01944-2 (PMC10247370; doi:10.1038/s41409-023-01944-2)
Supplement: Supplementary file 2 — Factors associated with time to death with recipient anti-CMV IgG group C (0-5 AU/ml) as reference group [file 41409_2023_1944_MOESM2_ESM.docx]

Supplementary Table: Factors associated with time to death with recipient anti-CMV IgG group C (0-5 AU/ml) as reference group

| Dependent Variable | a. Cox proportional−hazards model up to 12 months | | b. Cox proportional−hazards model up to 36 months | |
| --- | --- | --- | --- | --- |
|  | Adjusted hazard ratio | p- value | Adjusted hazard ratio | p- value |
| Age in years | 1.01 (1.00, 1.03) | 0.10 | 1.01 (0.99, 1.02) | 0.371 |
| Recipient anti−CMV IgG group  C (0−5 AU/ml)  B (6−249 AU/ml)  A (=250 AU/ml) | 1  0.99 (0.61, 1.63)  1.29 (0.68, 2.44) | 0.98  0.43 | 1  0.92 (0.63, 1.35)  1.60 (0.99, 2.58) | 0.682  0.053 |
| Donor CMV serostatus  Positive  negative | 1  1.22 (0.77, 1.92) | 0.40 | 1  1.30 (0.91, 1.85) | 0.144 |
| Disease risk  Low  Intermediate  High  very high | 1  1.18 (0.56, 2.49)  2.29 (1.09, 4.80)  2.85 (1.19, 6.85) | 0.66  0.03  0.02 | 1  1.58 (0.88, 2.84)  2.54 (1.41, 4.58)  2.78 (1.35, 5.76) | 0.122  0.002  0.006 |
| Match  Match  Mismatch | 1  1.33 (0.83, 2.13) | 0.23 | 1  1.16 (0.80, 1.68) | 0.436 |
| Conditioning  MAC  NMA  RIC | 1  1.63 (0.53, 5.03)  1.19 (0.68, 2.09) | 0.40  0.54 | 1  2.01 (0.85, 4.77)  1.43 (0.91, 2.24) | 0.113  0.123 |

MAC – myeloablative conditioning; NMA – non-myeloablative; RIC – reduced intensity conditioning; subCMVi – subclinical CMV infection; csCMVi – clinically significant CMV infection
